# Evaluation of large language models in assigning PI-RADS v2.1 categories for prostate MRI reports

**DOI:** 10.1186/s12894-025-02038-5

**Published:** 2026-01-02

**Authors:** Betul Akdal Dolek, Muhammed Said Besler

**Affiliations:** 1 Department of Radiology, Ankara Bilkent City Hospital, Ankara, Turkey; 2 Department of Radiology, İstanbul Medeniyet University Faculty of Medicine, Istanbul, Turkey

**Keywords:** Artificial intelligence, Large language model, Prostate imaging, Magnetic resonance imaging, Prostate cancer, Urology

## Abstract

**Background:**

This study aimed to evaluate the performance of large language models (LLMs) in classifying prostate MRI reports according to the Prostate Imaging–Reporting and Data System (PIRADS) version 2.1, and to validate their use in supporting clinical decisions in prostate cancer treatment.

**Methods:**

This retrospective study included 146 patients. Four LLMs — GPT-4o, GPT-o1, Google Gemini 1.5 Pro and Google Gemini 2.0 Experimental Advanced — were tested on standardised, structured prostate MRI reports. A two-radiologist consensus reference standard was used to compare model performance. Agreement was measured using weighted Cohen’s kappa, and accuracy and F1 scores were calculated for three PI-RADS risk groups: low (1–2), intermediate (3) and high (4–5).

**Results:**

Performance varied by model. GPT-o1 achieved the highest level of agreement with radiologists (κ = 0.867), followed by GPT-4o (κ = 0.743), Gemini 1.5 Pro (κ = 0.728) and Gemini 2.0 Experimental Advanced (κ = 0.664). GPT-o1 achieved the highest F1 scores for the low-risk (0.93) and high-risk (1.00) groups, demonstrating moderate performance for the PI-RADS 3 group (0.75). All models showed weak performance for PI-RADS 3 (F1 range: 0.54–0.75). Most importantly, none of the models produced invalid results outside the target PI-RADS 1–5 range.

**Conclusion:**

LLMs show potential for automating PI-RADS classification from MRI reports, with GPT-o1 demonstrating the best overall performance. However, their failure in PI-RADS 3 lesions indicates that multicentre validation, larger datasets and multimodality integration are needed before they can be used clinically for prostate cancer diagnosis and urological decision-making.

**Trial registration:**

Not applicable. This retrospective study did not involve a clinical trial.

## Introduction

Prostate cancer is one of the leading causes of cancer-related illness and death among men worldwide. This necessitates accurate diagnostic methods for appropriate risk stratification [1, 2]. The Prostate Imaging-Reporting and Data System (PI-RADS) version 2.1 is a standardized system for reporting prostate magnetic resonance imaging (MRI) that aids in the detection and risk stratification of clinically significant prostate cancer and directly influences urological decision-making [3]. Beyond radiology, PI-RADS has become an important decision-support tool in urology, directly impacting biopsy recommendations, treatment plans and follow-up strategies. PI-RADS 3 represents the most challenging category, as its equivocal nature may lead to unnecessary biopsies or delayed diagnosis, with important clinical implications.

Recent advances in artificial intelligence (AI) and large language models (LLMs), such as OpenAI’s Generative Pre-trained Transformer (GPT) and Google’s Gemini, have sparked significant optimism regarding their potential to automate and standardise radiological reporting procedures [4–7].

Several recent studies have observed the use of LLMs for *RADS classification, such as breast imaging with the BI-RADS system, lung cancer screening with Lung-RADS and liver imaging with LI-RADS [8–11].

Identifying PI-RADS categories directly from MRI reports through generative AI could potentially streamline clinical workflows, ensure reporting consistency, and reduce interobserver variability. Although Lee et al. demonstrated the feasibility of LLM-based PI-RADS classification, their study was limited by a small sample size and earlier-generation models [12]. In contrast, our work involved newer generation LLMs from a larger patient population. Furthermore, our study was conducted at a different institution, providing external validation and enhancing the generalisability of the results to various clinical settings.

This study aimed to evaluate the performance of state-of-the-art LLMs in assigning PI-RADS v2.1 categories from structured prostate MRI reports, using expert radiologist consensus as the reference standard. By examining diagnostic accuracy, error patterns, and model-driven biases, we intend to enhance our comprehension of the clinical application of these tools and their potential influence on urological management pathways.

## Methods

### Study design and acquisition of data

**Fig. 1:**
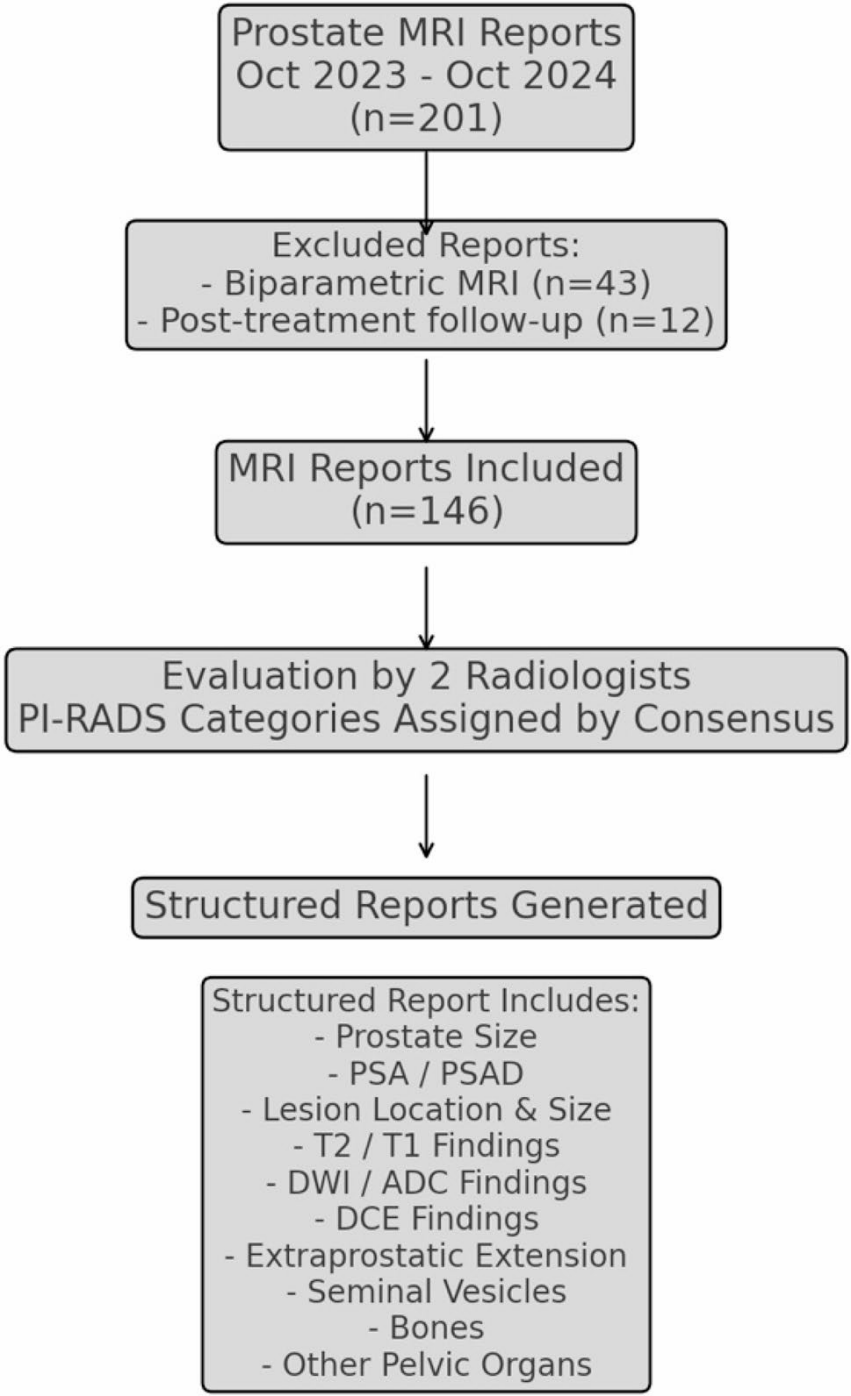
Study design. DCE: dynamic contrast enhanced images; PI-RADS: Prostate Imaging- Reporting and Data System; PSA: prostate specific antigen; PSA density: prostate specific antigen density

This retrospective analysis was done under the ethical standards and with the institutional review board approval, which waived informed consent because of anonymized data. Prostate MRI reports were collected in a single tertiary care center between October 2023 and October 2024. Biparametric MRI examinations and MRIs of post-treatment follow-up patients were excluded. 146 reports were included in the study (Fig. 1).

A radiologist with 5 years of experience in prostate MRI and a uroradiologist with 10 years of experience independently reviewed prostate MRI reports, which were structured in a standardized format and authored by different radiologists in the clinic. They assigned PI-RADS categories by consensus.

Reports written during this period alone were utilized to maintain consistency in imaging protocols and adherence to the latest PI-RADS v2.1 guidelines [3]. Clinical history and PI-RADS categories were removed from reports to present a blinded and standardized assessment process. All reports were structured similarly, containing detailed descriptions of T2-weighted imaging (T2WI), diffusion-weighted imaging (DWI) with ADC values, dynamic contrast-enhanced (DCE) apperances, prostate volume, PSA density and lesion size (Fig. 1). Structured MRI reports included numerical PSA values (ng/mL) and PSA density values (ng/mL/cm³) presented in a dedicated clinical information section. Descriptive qualifiers such as ‘high PSA’ were not used. These parameters were provided as contextual clinical data and were not formally incorporated into PI-RADS scoring by radiologists.

### Large language models and assessment protocol

The LLMs evaluated in this study included GPT-4o, GPT-o1, Google Gemini 1.5 Pro, and Google Gemini 2.0 Experimental Advanced.

In December 2024, all models were given the same standardised prompt to ensure consistency in task interpretation: You are an expert diagnostic radiologist. Evaluate the following prostate MRI report in strict accordance with PI-RADS v2.1 guidelines, assigning a definitive PI-RADS category (1–5). Avoid speculation and provide a clear justification based solely on the given information. Do not assume medico-legal responsibility. All GPT-based models were accessed using their default automatic response mode without enabling extended reasoning or step-by-step explanation features. Model outputs reflected a single-pass response to the standardized prompt, without iterative refinement or user feedback.

MRI reports were sequentially input to each model in randomized order to eliminate potential bias. The order was neither categorized by PI-RADS category nor influenced by clinical variables. Findings were recorded separately. MRI reports were uploaded as plain text files (.txt) to ensure uniformity across the models. To ensure reproducibility, all prompts and interactions were logged, and results were independently verified by two researchers.

### Statistical analysis

SPSS 26.0 was used to perform statistical analyses (IBM Corp., Armonk, NY, USA). Inter-rater agreement between the LLM-assigned PI-RADS categories and the gold standard was quantified using weighted Cohen’s Kappa, which accounts for the ordinal nature of the PI-RADS scoring system. The following criteria were used to interpret the strength of agreement: <0.20 (poor), 0.21–0.40 (fair), 0.41–0.60 (moderate), 0.61–0.80 (substantial), and > 0.80 (almost perfect) [13].

Accuracy and F1 scores were derived for three stratified PI-RADS risk groups: low-risk (1–2), intermediate-risk (3) and high-risk (4–5).

## Results

The data included MRI reports labelled according to PI-RADS, with 22, 53, 28, 25 and 18 reports for categories 1, 2, 3, 4 and 5 respectively. Concordance between the categories assigned by the models and the gold standard varied among the LLMs tested. As shown in Table 1, GPT-o1 achieved the highest overall agreement, with a weighted Cohen’s kappa of 0.867 (*p* < 0.001). This was followed by GPT-4o (κ = 0.743, *p* < 0.001), Google Gemini 1.5 Pro (κ = 0.728, *p* < 0.001) and Google Gemini 2.0 Experimental Advanced (κ = 0.664, *p* < 0.001). GPT-o1 performed best among the other models in terms of accuracy and F1 performance by PI-RADS risk category.

**Table 1:** Performance metrics of large Language models in assigning PI-RADS categories

Model	PI-RADS 1–2 Accuracy (%)	PI-RADS 3 Accuracy (%)	PI-RADS 4–5 Accuracy (%)	PI-RADS 1–2 F1 Score	PI-RADS 3 F1 Score	PI-RADS 4–5 F1 Score	Weighted Kappa
GPT-o1	93.3	75	100	0,93	0,75	1	0,867
GPT-4o	92	53.6	90.7	0,92	0,54	0,91	0,743
Google Gemini 1.5 Pro	93.3	53.6	86	0,93	0,54	0,86	0,728
Google Gemini 2.0 Exp. Advanced	81.3	57.1	88.4	0,81	0,57	0,88	0,664

**Fig. 2:**
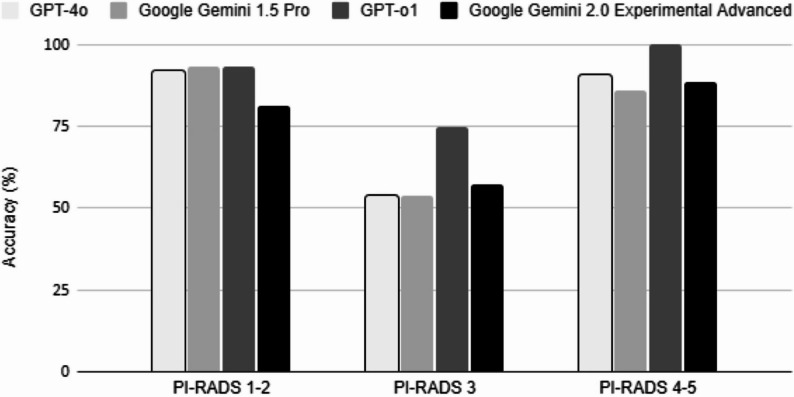
Comparison of accuracy (%) among large language models (GPT-4o, Google Gemini 1.5 Pro, GPT-o1, and Google Gemini 2.0 Experimental Advanced) across different PI-RADS v2.1 categories (PI-RADS 1–2: low-risk, PI-RADS 3: intermediate-risk, PI-RADS 4–5: high-risk)

For low-risk cases (PI-RADS 1–2), GPT-o1 reached 93.3% accuracy (95% CI: 81.7%–98.6%) and an F1 score of 0.93, followed by Google Gemini 1.5 Pro (accuracy: 93.3%, 95% CI: 81.7%–98.6%, F1: 0.93), GPT-4o (accuracy: 92.0%, 95% CI: 79.6%–97.6%, F1: 0.92), and Gemini 2.0 (accuracy: 81.3%, 95% CI: 67.4%–90.3%, F1: 0.81) (Fig. 2). GPT-4o misclassified 2.7% of these cases as high-risk (PI-RADS 4–5), while other models had a lower misclassification rate of 1.3%.

Performance dropped notably across all models in intermediate-risk cases (PI-RADS 3). GPT-o1 achieved the highest accuracy (accuracy: 75.0%, 95% CI: 55.1%–89.3%) and an F1 score of 0.75, whereas Google Gemini 2.0 Experimental Advanced reached 57.1% accuracy (95% CI: 37.2%–75.5%, F1: 0.57), and both GPT-4o and Gemini 1.5 Pro recorded 53.6% accuracy (95% CI: 33.9%–72.5%) and an F1 score of 0.54.

In the high-risk group (PI-RADS 4–5), GPT-o1 performed best once more, achieving 100% accuracy (95% CI: 86.3%–100%) and an F1 score of 1.00. Next was GPT-4o with 90.7% accuracy (95% CI: 73.8%–97.5%, F1 score: 0.91), followed by Gemini 2.0 with 88.4% accuracy (95% CI: 70.0%–96.4%, F1 score: 0.88), and then Gemini 1.5 Pro with 86.0% accuracy (95% CI: 67.0%–95.5%, F1 score: 0.86). Only Gemini 1.5 Pro misclassified 4.7% of high-risk reports as low-risk (PI-RADS 1–2), an error with potential clinical significance.

In addition to accuracy, sensitivity and specificity metrics with 95% CI were calculated for each model across the three PI-RADS risk categories (1–2, 3, and 4–5). GPT-o1 showed the most balanced overall performance:

- Low-risk (PI-RADS 1–2): Sensitivity 93.3% (95% CI: 81.7–98.6%), Specificity 100% (95% CI: 94.6–100%).
- Intermediate-risk (PI-RADS 3): Sensitivity 75.0% (95% CI: 55.1–89.3%), Specificity 84.0% (95% CI: 73.3–91.8%).
- High-risk (PI-RADS 4–5): Sensitivity 100% (95% CI: 86.3–100%), Specificity 84.0% (95% CI: 68.9–93.0%).

GPT-4o followed with:

- Low-risk: Sensitivity 92.0% (95% CI: 79.6–98.4%), Specificity 95.8% (95% CI: 89.6–98.9%).
- Intermediate-risk: Sensitivity 53.6% (95% CI: 33.9–72.5%), Specificity 65.2% (95% CI: 52.4–76.5%).
- High-risk: Sensitivity 90.5% (95% CI: 77.4–97.3%), Specificity 76.0% (95% CI: 60.6–87.9%).

Google Gemini 1.5 Pro demonstrated:

- Low-risk: Sensitivity 93.3% (95% CI: 81.7–98.6%), Specificity 93.8% (95% CI: 86.0–98.0%).
- Intermediate-risk: Sensitivity 53.6% (95% CI: 33.9–72.5%), Specificity 65.2% (95% CI: 52.4–76.5%).
- High-risk: Sensitivity 86.0% (95% CI: 71.3–94.2%), Specificity 72.0% (95% CI: 56.3–84.7%).

Google Gemini 2.0 Experimental Advanced showed the lowest overall performance:

- Low-risk: Sensitivity 81.3% (95% CI: 67.4–91.1%), Specificity 85.4% (95% CI: 75.7–92.3%).
- Intermediate-risk: Sensitivity 57.1% (95% CI: 36.5–75.5%), Specificity 76.8% (95% CI: 65.4–85.8%).
- High-risk: Sensitivity 88.4% (95% CI: 74.9–96.1%), Specificity 68.0% (95% CI: 52.4–81.4%).

A notable observation was the tendency of Google Gemini 2.0 Experimental Advanced to upgrade PI-RADS 1–2 cases to PI-RADS 3, particularly when prostate-specific antigen (PSA) levels were elevated and moderate diffusion restriction was present. Nevertheless, none of the models produced invalid outputs outside the defined PI-RADS 1–5 range.

## Discussion

The findings of this study highlight the variable diagnostic accuracy of LLMs in assigning PI-RADS categories from text-based prostate MRI reports. Among all of the models evaluated, GPT-o1 had the highest diagnostic agreement with radiologists, with a weighted Cohen’s Kappa of 0.867 and higher F1 scores in all PI-RADS categories: 0.93 (PI-RADS 1–2), 0.75 (PI-RADS 3), and 1.00 (PI-RADS 4–5). These results suggest that GPT-o1 not only achieved high concordance with radiologists overall but also maintained consistent precision and recall across strata of risk. By contrast, GPT-4o and Google Gemini 1.5 Pro, while very good in low-risk categories (F1 = 0.92 and 0.93 respectively), exhibited limited effectiveness in intermediate-risk lesions (both F1 = 0.54). These misclassifications can have significant clinical repercussions. For instance, upgrading from PI-RADS 1–2 to 4–5 due to false-positive results can lead to unnecessary biopsies, increased healthcare expenditure, and anxiety in patients [14, 15]. An accurate PI-RADS classification is crucial for making the right decision about whether to perform a biopsy. Misclassifying low-risk lesions as high-risk can lead to unnecessary procedures, while misclassifying high-risk lesions as low-risk can result in a delayed diagnosis of clinically relevant prostate cancer. GPT-4o and Gemini 1.5 Pro have false-positive rates of 2.7% and 4.7%, respectively. These over classifications are probably due to the models’ inability to distinguish subtle imaging characteristics, such as benign focal diffusion restriction and mild T2 hypointense, which are common in benign prostatic hyperplasia.

Notably, Google Gemini 2.0 Experimental Advanced had the weakest overall agreement (Kappa = 0.664) and the lowest F1 performance in low-risk lesions (F1 = 0.81). The model showed a clear tendency to upgrade PI-RADS 1–2 cases to PI-RADS 3, particularly in cases of high PSA density and moderate diffusion restriction. Although PSA is not an official component of the PI-RADS v2.1 scoring system, its incorporation into structured reports is recommended for providing clinical context [3]. Although PSA is a key marker for prostate cancer screening, its use in PI-RADS classification is controversial due to the risk of overdiagnosis and overtreatment of indolent lesions [16–19]. Conversely, Gemini 1.5 Pro occasionally downgraded some high-risk (PI-RADS 4–5) lesions to low-risk lesions. These biases most likely reflect elements of the models’ training data or prompt interpretation.

Overall, underperformance was observed for PI-RADS 3 lesions, with F1 scores ranging from 0.54 to 0.75 across all models. Underperformance in this category is to be expected due to its inherent ambiguity, which is always associated with the lowest level of agreement among radiologists [20]. Typical misclassified PI-RADS 3 reports included statements such as mild diffusion restriction without corresponding low ADC values, or focal T2 hypointensity with equivocal enhancement on DCE. In these cases, models frequently upgraded lesions to PI-RADS 4, particularly when PSA density values were elevated. Qualitative analysis of misclassified instances revealed recurring failure modes. The models tended to overestimate PSA values or misinterpret descriptive phrases such as ‘equivocal findings’ or ‘borderline restriction’. In some cases, there was a tendency to upgrade PI-RADS 3 reports to higher categories if PSA levels were elevated. Conversely, equivocal diffusion findings were sometimes downgraded, which could lead to a delayed diagnosis. These results suggest that LLMs rely too heavily on contextual clinical information and linguistic cues, rather than strictly adhering to imaging-based language. This issue could be addressed through advanced prompt engineering, providing explicit instructions to ignore PSA when assigning categories, or fine-tuning using domain-specific corpora [12, 21].

Despite these limitations, LLMs are set to be integrated into radiology workflows. Applications include summarizing key findings [22, 23],, answering image-related questions [24], converting free text into structured reports [25, 26] and offering differential diagnoses [27, 28]. However, LLMs are known to generate false or misleading information through internal reasoning, a phenomenon commonly referred to as ‘hallucinations’ [4, 29–31]. The Lee et al. experiment showed that Bard produced irrelevant categories, such as PI-RADS 6 [12]. However, none of the models we used produced outputs outside the valid range of PI-RADS 1–5. This demonstrates continued consistency with the diagnostic system, which is clinically relevant.

This study has some limitations. Firstly, the single-centre retrospective design may restrict generalisability. Variation in terminology, reporting style and imaging protocols across institutions may affect LLM performance. Therefore, multicentre studies are required in future to determine robustness in different clinical settings and reduce institutional bias. Secondly, the small sample size (*n* = 146) and the unbalanced distribution of categories, particularly for PI-RADS 5, may limit statistical power. Future analyses would be improved by more datasets and stratified sampling. Thirdly, the study focused only on text reports of MRI scans, not the imaging data itself. Multimodal AI approaches that integrate imaging features with text-based data are likely to improve performance, particularly for indeterminate PI-RADS 3 cases.

In conclusion, while LLMs are not yet ready to complement radiologists, they could serve as helpful decision aids in prostate MRI interpretation. Addressing their current limitations through multicentre validation, larger and more representative datasets, and multimodality integration is the key to ensuring their safe, accurate, and clinically effective application in prostate cancer diagnosis.

## Data Availability

The data that support the findings of this study are available on request from the corresponding author.

## Abbreviations

AI: Artificial Intelligence
ADC: Apparent Diffusion Coefficient
BI: RADS–Breast Imaging Reporting and Data System
DCE: Dynamic Contrast–Enhanced
DWI: Diffusion–Weighted Imaging
F1: F1 Score
GPT: Generative Pre–trained Transformer
Kappa (κ): Cohen’s Kappa Coefficient
LI: RADS–Liver Imaging Reporting and Data System
LLM: Large Language Model
Lung: RADS–Lung Imaging Reporting and Data System
MRI: Magnetic Resonance Imaging
PI: RADS–Prostate Imaging Reporting and Data System
PSA: Prostate–Specific Antigen
PSAD: Prostate–Specific Antigen Density
